# Different Effects of Robot-Assisted Gait and Independent Over-Ground Gait on Foot Plantar Pressure in Incomplete Spinal Cord Injury: A Preliminary Study

**DOI:** 10.3390/ijerph182212072

**Published:** 2021-11-17

**Authors:** Young-Hyeon Bae, Won Hyuk Chang, Shirley S. M. Fong

**Affiliations:** 1 Korea National Rehabilitation Center, Department of Healthcare and Public Health, Rehabilitation Research Institute, Seoul 01022, Korea; 2 Center for Prevention and Rehabilitation, Department of Physical and Rehabilitation Medicine, Heart Vascular Stroke Institute, Sungkyunkwan University School of Medicine, Seoul 06351, Korea; iamchangwh@naver.com; 3 Li Ka Shing Faculty of Medicine, School of Public Health, University of Hong Kong, Hong-Kong 999077, China; smfong@yahoo.com.hk

**Keywords:** spinal cord injury, rehabilitation, gait, robot-assisted, foot plantar pressure

## Abstract

Background: There is insufficient evidence to establish the optimal treatment protocol for robot-assisted gait training. Objective: This study aimed to analyze the effects of robot-assisted gait and independent over-ground gait on foot pressure and to determine an effective training protocol for improvement of gait pattern in patients with incomplete spinal cord injury due to industrial accidents. Methods: Four patients with incomplete spinal cord injury due to an industrial accident who had gait disturbance underwent measurement of peak foot pressure and stance phase duration using a foot pressure analysis system with robot-assisted gait and independent over-ground gait. Results: The robot-assisted gait condition has lower peak foot pressure and shorter stance phase duration than the independent over-ground gait. Conclusions: In this study, robot-assisted gait was found to limit gait pattern improvement in patients with gait disturbance caused by incomplete spinal cord injury due to industrial accidents. Therefore, future research will be conducted to determine the optimal protocol for robot-assisted gait training for gait pattern improvement.

## 1. Introduction

Patients with gait disturbance require excessive physical effort of three or more skilled physiotherapists to repeatedly perform safe gait training by imitating normal gait patterns. Moreover, it is almost impossible for patients with severe muscle weakness or involuntary motions to perform gait training even with the help of therapists [1,2,3,4].

The Body-Weight Support Treadmill Training (BWSTT) is a training method based on normal physiological gait patterns with a focus on the temporal aspect and ideal kinematics of walking. Activity-dependent neural plasticity observed in animal research has been applied as a basic treatment concept for BWSTT in patients with early spinal cord injury [1,2,3,4]. This gait training method has been applied in patients with other types of nerve injury, such as stroke and Parkinson’s disease [1,2,3,4,5]. A previous study reported that speed dependent BWSTT was especially effective for patients with stroke; hence, attention was placed on the use of BWSTT as a stroke gait training method [1]. However, problems were encountered due to the limitations of BWSTT in terms of persistence and consistency of sensory stimuli [6]. To overcome these limitations, the treatment principles of the BWSTT have been digitally programmed [6]. Furthermore, a robot-assisted gait (RAG) device has been developed to reduce the physical effort and time of therapists, and to increase the repetitive walking time of gait kinematics. The RAG device is designed to induce the movements of patient’s lower limbs according to pre-programmed normal gait patterns and is being applied in the treatment of patients with gait disturbance. The RAG training paradigm offers intensive, repetitive, accurate kinematic feedback and symmetrical gait practice, while reducing the workload for the therapist, reducing the cost of rehabilitation [2,5,6,7,8,9,10,11,12].

To restore gait, two innovative robot-assisted gait device paradigms utilizing the end-effector and exoskeleton robot types have recently been adopted to provide an ample number of repetitions with precise kinematics and kinetic sensorimotor feedback. Numerous RAG devices have been developed and commercialized, including the Lokomat, Walkbot, E-GO, and ReoAmbulator. Despite their commercial availability, these products still have several limitations, the most important being the inability to support over-ground gait [2,5,6,7,8,9,10,11,12].

The RAG was initially studied in acute and sub-acute phases and has recently been reported to be effective in the chronic phase of neurological disease [13]. The RAG is beneficial for normalizing muscle tone, restoring functional walking, and improving lower extremity function, which may enable spinal cord injury (SCI) patients to maintain a healthy lifestyle and increase their level of physical activity [14,15]. However, most studies have reported that its effects are comparable to those of traditional gait training. With regard to the reasons why the RAG device failed to show excellent effects, previous studies reported that it has limitations in inducing normal movements, such as the abnormal sensory stimuli created by a strap used to fix the patient’s lower limbs to the robot, lower limb movements that only occur in the sagittal plane, passively induced movements, lack of movements of the pelvis and trunk, and absence of an effective weight shift [6].

To analyze the effects of the RAG on gait improvement, differences in foot pressure distribution during walking between RAG training and BWSTT were studied [16]. It was also found that BWSTT and RAG training do not increase walking speed more than overground gait training and other forms of physiotherapy, but their effects on walking distance are not clear [17]. Therefore, almost no research has been conducted to determine the differences in gait between the RAG condition, which moves by active assist under the help of a robot, and the active independent over-ground gait (IOGG) condition. Because the additive effect of robot-assisted gait training is well established, a prospective study is warranted to determine if robot-assisted gait training is superior to conventional locomotor rehabilitation alone or robot-assisted gait training combined with conventional gait training. This study aimed to analyze the effects of the RAG and IOGG on foot pressure and to determine an effective training protocol for improvement of gait pattern in patients with incomplete spinal cord injury due to industrial accidents.

## 2. Methods

### 2.1. Patients

A preliminary study was conducted including four patients with incomplete spinal cord injuries who had gait disturbance and were undergoing RAG training in a hospital specializing in industrial accidents. All patients had partial paralysis of the lower body and were men.

Inclusion criteria were as follows: patients who had an industrial accident resulting in gait disturbance, can walk independently using assistive devices, have no visual defects, can maintain stable stance during examination, can follow instructions, and have stable medical status. Exclusion criteria were as follows: excessive weight gain (135 kg or more) when the RAG is applied and no identified causes of weight load on the lower limbs such as fracture, skin injuries or bedsores, cardiovascular diseases or heart failure, malignant diseases, lung diseases, neurological diseases, or other underlying diseases that prevent the use of the RAG. The written consent of participants was obtained after explaining the study, and the study was approved by the institutional review board.

### 2.2. Study Procedure

The foot pressure distribution changes of the four selected patients were measured in the stance phase of the gait cycle using a foot pressure analysis system instrument in both IOGG and RAG conditions, with an interval of one day between measurements. In the IOGG condition, foot pressure distribution was measured with a 10 m walking test on the ground with usual walking aids, and in the RAG condition, foot pressure distribution was measured with RAG training. Then, the gait patterns between the two gait conditions were compared based on the changes in the foot pressure distribution. The distribution of plantar pressure was quantified in standing and gait using a pressure measurement device.

### 2.3. IOGG

**Figure 1:**
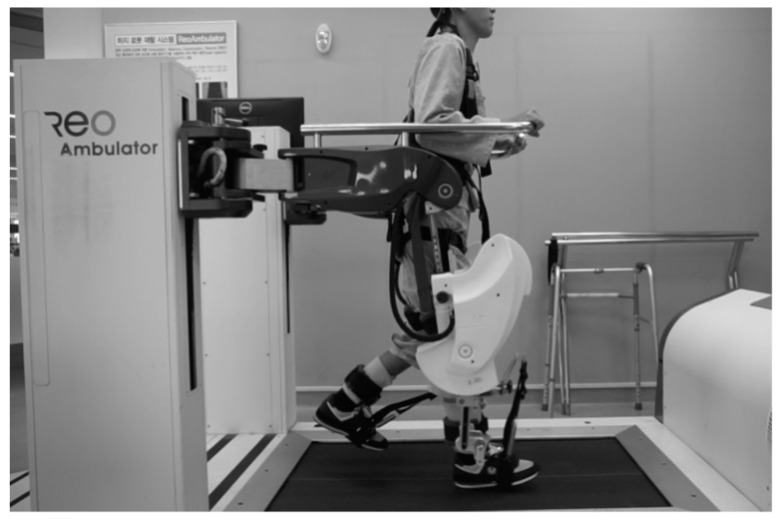
Robot assisted gait.

A 10 m walking test was performed to analyze the IOGG. The 10 m walking test measures the duration of walking a 10 m distance by independent walking regardless of the existence or absence of assistive devices. Although the study participants could walk independently, the therapists continuously monitored them at a close distance to prepare for possible safety-related accidents. Gait analysis was performed using a foot pressure analysis system. The mean speed in the 10 m walking test was 1.14 ± 0.15 m/s (Figure 1) [18].

### 2.4. Robot-Assisted Gait

**Figure 2:**
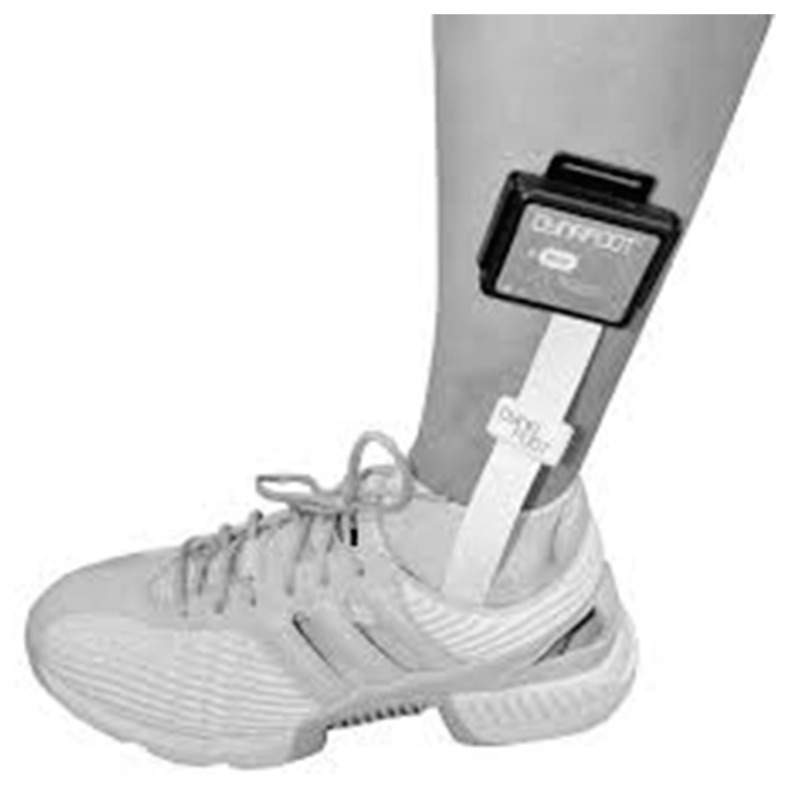
Foot pressure analysis system.

The ReoAmbulator (ReoAmbulator^TM^, Motorika USA Inc., Mount Laurel, NJ, USA) device consists of a RAG device for attitude control and a body-weight support device and works by interlinking with a treadmill (Figure 2) [19]. The RAG training process is as follows. The patient wears a harness attached to the body-weight support device, which supports the patient so that the patient can safely stand on a treadmill. The attitude controller is a computer-controlled robot device designed to fit the patient’s hip and knee joints and adjust the joint motions in line with the walking speed. Once the treadmill is started with the patient lifted with a body-weight support device and a RAG device, the patient is lowered to the treadmill, and RAG training programmed to the normal physiological gait pattern begins. Depending on the gait pattern, the therapist can appropriately adjust the treadmill’s speed, joint movement speed, and angle through a computer, and the body-weight support on the body-weight support device.

The speed, guidance force, and body-weight support during the RAG training were adjusted based on the therapist’s decision in accordance with the individual conditions of the patients to achieve patient comfort. In this study, the RAG device’s speed was adjusted to the same as the 10 m walking speed, with IOGG, guidance force, and body-weight support were set to a minimum.

### 2.5. Measures

We extracted information regarding American Spinal Injury Association Impairment Scale (AIS) grade (A, B, C and D), height, weight, age, duration of injury (DoI), lower extremity motor scale (LEMS), level of injury (LoI) obtained during acute in-hospital stay. Maximum and minimum LEMS were 50 and 0, respectively. The peak foot pressure and stance phase duration during the RAG and IOGG conditions were measured using a foot pressure analysis system (Dynafoot2, Techno concept, Saint-Maurice, France) [20]. Under the RAG condition, the average of three steps of both feet with the patient and therapists in optimal training condition was used as the analysis data, whereas under the IOGG condition, the average of three steps of both feet after the third step in the 10 m walking test was used as the analysis data.

### 2.6. Data Analysis

In this study, the general characteristics of patients are reported as means and standard deviations. Wilcoxon’s signed-rank test was used to analyze the differences in foot pressure distribution data, including peak foot pressure and stance phase duration between the two gait conditions. For statistical analysis, the SPSS 24.0 software (IBM SPSS Inc., Armonk, NY, USA) was used, and the significance level was set at lower than 0.05.

## 3. Results

The participants’ mean height was 175.5 ± 5.7 cm, mean weight was 65.6 ± 12.2 kg, mean age was 42.3 ± 15.2 years, mean DoI was 370.8 ± 11.0 days, and mean LEFS was 12.8 ± 3.3. Regarding damage levels, two patients had an injury to the 12th thoracic vertebra and two patients had an injury to the 1st lumbar vertebra (Table 1).

The RAG had lower peak foot pressure than IOGG in the strong and weak legs. Moreover, the RAG had a shorter stance phase duration than IOGG in the strong and weak legs. However, all variables were not significantly different between the two conditions (Table 2).

## 4. Discussion

The RAG device has been developed to reduce the physical effort and time of therapists, and to increase the repetitive walking time of gait kinematics. The RAG device is designed to induce the movements of patient’s lower limbs according to pre-programmed normal gait patterns and is being applied in the treatment of patients with gait disturbance. The RAG device provides a more supportive environment and normalized physiological gait training with benefits of temporal aspects and ideal kinematics [2,5,6,7,8,9,10,11,12,13,14,15].

RAG training, when applied with a general rehabilitation program, has been reported to slightly improve the mobility of patients with sub-acute stroke or spinal cord injury, and to be more effective in improving functional motions when applied with longer duration and higher intensity. However, evidence to establish the optimal treatment protocol is insufficient [7,10,11,21,22].

Despite this situation, RAG training has been applied recently for patients with various gait disturbances [8]. However, the evidence is insufficient, and the results are inconsistent for evaluation of the effects of RAG training on patients with cerebral palsy because only the speed, standing ability, and distance have been evaluated [8]. In the analysis of the effect of RAG training on patients with traumatic brain injury, only the independent gait speed and maximum speed on the ground increased, with no improvement in symmetrical gait [8]. Furthermore, in patients with Parkinson’s disease, no significant difference was found in the 6 min walking test and the 10 m walking test between RAG training and BWSTT [5]. The application of RAG training after cardiac surgery showed an advantage over general rehabilitation methods only in walking distance [9]. Thus, although RAG training has been applied in the treatment of various diseases, including cerebral palsy, Parkinson’s disease, and myocardial infarction, most reports are case studies, and conclusive evidence concerning the therapeutic effects is inadequate [2]. As such, studies to determine the optimal protocol design and to establish the long-term effects of RAG training in neurological diseases are required [2].

This study was conducted to analyze the difference between the RAG and IOGG on foot pressure distribution and determine the effective training protocol for improvement of the gait pattern in incomplete spinal cord injury with an asymmetrical muscle imbalance.

The results of this study showed that the RAG had lower peak foot pressure and shorter stance phase duration compared to the IOGG.

In previous studies, the guidance force of RAG training decreased the activities of muscles that produce stability and propulsive force, such as the erector spinae, gluteus medius, biceps femoris, and medial gastrocnemius [23]. Increased body-weight support decreased the anteroposterior and lateral displacements of the pelvis and trunk, and the axial rotation and anteroposterior flexion of the trunk, but the lateral flexion of the trunk and anteroposterior pelvic inclination increased [12]. As the guidance force increased, the maximum displacements of the pelvis and trunk decreased, whereas the posterior pelvic inclination increased [10,11]. Furthermore, ankle immobilization by ankle straps has been reported to limit gait improvement [7]. Therefore, RAG training does not increase walking speed more than overground gait training and other forms of physiotherapy, but their effects on walking distance are not clear [17].

As outlined above, previous studies reported that RAG training increased the anteroposterior pelvic inclinations of the pelvis, decreased most of the trunk and pelvic movements, and made normal gait pattern training difficult due to ankle immobilization. In the present study, various problems were discovered, such as arbitrary control and intensity setting of the RAG by the therapists and patients, ankle immobilization during RAG training, difficulty of adjusting RAG speed, forward tilting of the trunk due to passive participation of patients, medial rotation pattern of the lower limbs, arm fixation, and limited normal gait patterns. Given these limitations, active gait training using the normal pattern could not be achieved. Hence, the RAG did not utilize the repulsive force of the ground sufficiently compared to IOGG, resulting in a low pressure of much less than 120% of the body weight, which is the peak foot pressure of normal people. In addition, an abnormal gait pattern occurred when the stance phase duration responding to the ground decreased due to foot immobilization. This study has limitations, in that we performed relative comparisons by adjusting the walking speed between the RAG and IOGG. However, the RAG was only controlled to minimize the effects of guidance force and body-weight support. Therefore, future research will be conducted to determine the optimal protocol for robot-assisted gait training for gait pattern improvement and the clinical usability of the types of RAG device. Furthermore, it is necessary to consider additional research on gait training while applying a wearable assistive limb robot on the ground.

## 5. Conclusions

Almost no research has been conducted to determine the differences in gait between the RAG condition, which moves by active assist under the help of a robot, and the active IOGG condition. This study aimed to analyze the effects of the RAG and IOGG on foot pressure and to determine an effective training protocol for improvement of gait pattern in patients with incomplete spinal cord injury due to industrial accidents. As result, the robot-assisted gait condition has lower peak foot pressure and shorter stance phase duration than the independent over-ground gait. However, this study has limitations, in that we performed relative comparisons by adjusting the walking speed between the RAG and IOGG, but the RAG was only controlled to minimize the effects of guidance force and body-weight support. Thus, the robot-assisted gait was found to limit gait pattern improvement in patients with gait disturbance caused by incomplete spinal cord injury due to industrial accidents. Therefore, in the future, an optimal protocol should be investigated through a systematic analysis to improve the walking of patients with gait disturbance using the RAG, and the equipment should be improved accordingly.

## Figures and Tables

**Table 1:** Characteristics of the study subjects.

Variable	Patient 1	Patient 2	Patient 3	Patient4	Mean ± SD
Age (year)	42	37	63	27	42.3 ± 15.2
Height (cm)	178	179	178	167	175.5 ± 5.7
Weight (kg)	69	63	80	50.7	65.6 ± 12.2
DoI (day)	454	307	249	473	370.8 ± 110.0
LEMS (Lt/Rt)	9 (5/4)	11 (6/5)	15 (9/6)	16 (9/7)	12.75 ± 3.30
LoI	T12	T12	L2	L1	
AIS (grade)	C	C	C	C	

DoI—duration of injury; LEMS—lower extremity motor scale; LoI—level of injury; AIS—American Spinal Injury Association Impairment Scale; T—Thoracic; L—Lumbar.

**Table 2:** Difference in foot pressure distribution between strong and weak legs.

Variable		RAG Condition	IOGG Condition	z	*p*
		Mean ± SD	Mean ± SD		
Peak foot pressure (kg)	Strong leg	43.01 ± 24.33	60.74 ± 22.85	−1.826	0.068
	Weak leg	29.24 ± 2.33	40.17 ± 4.85	−1.826	0.068
Stance phase Duration (s)	Strong leg	1.22 ± 0.20	1.36 ± 0.36	−0.730	0.465
	Weak leg	0.99 ± 0.06	1.25 ± 0.45	−1.069	0.285

IOGG—independent over-ground gait; RAG—robot-assisted gait; All variables were not significantly different between the two conditions; Wilcoxon signed-rank test, *p* < 0.05.

## Data Availability

The data used and/or analyzed during the current study are available from the corresponding author on request.
